# Aerogel for Highly Efficient Photocatalytic Degradation

**DOI:** 10.3390/gels10020100

**Published:** 2024-01-26

**Authors:** Xue-Chun Yang, Jing-Tai Zhao

**Affiliations:** 1 School of Environmental and Chemical Engineering, Shanghai University, Shanghai 200444, China; xuechunyang@i.shu.edu.cn; 2 School of Materials Science and Engineering, Guilin University of Electronic Technology, Guilin 541004, China

**Keywords:** g-C_3_N_4_, carbon dots, agar, aerogel, amoxicillin

## Abstract

Photocatalysis is one of the effective ways to degrade pollutant antibiotics. Agar is used as the adsorption module to provide abundant pore structure. Carbon dots (CDs) are selected as light energy conversion components. Graphitic carbon nitride (g-C_3_N_4_) is used as the main material of the catalyst. Agar/CDs/g-C_3_N_4_-functionalized aerogel with a unique 3D pore structure is assembled. The Agar/CDs/g-C_3_N_4_ aerogel shows the highest photocurrent density, which is 3.7 times that of agar, 2.4 times that of 3-g-C_3_N_4_ and 1.6 times that of Agar/g-C_3_N_4_ aerogel. Compared with 3-g-C_3_N_4_ and Agar/g-C_3_N_4_ aerogel, which can completely remove AMX after 75 min, Agar/CDs/g-C_3_N_4_ aerogel can degrade amoxicillin (AMX) completely after 45 min of illumination. The reason is that Agar/CDs/g-C_3_N_4_ aerogel has a larger specific surface area, richer functional groups, a wider spectral range, higher photocurrent density and better carrier migration and separation efficiency. It is a good strategy with which to combine the effects of each component in the ternary system for the efficient photocatalysis of organic pollutants.

## 1. Introduction

Amoxicillin (AMX) as a β–Lactam antibiotic has a wide range of applications in medicine and veterinary medicine [1]. AMX has been found in groundwater, surface water, and soil, which pose serious risks, including human endocrine disorders and antibiotic resistance [2,3,4]. Due to its complex structure, high chemical stability and good solubility in aquatic environments, it is challenging to remove AMX from wastewater using traditional wastewater treatment plants. Therefore, the advanced oxidation process (AOP) is an effective and promising technology for purifying various water pollutants, but it is limited by the high cost of intensive chemical inputs and post-treatment.

Photocatalysis technology is considered one of the most attractive and promising technologies to achieve green production. High-efficiency photocatalysts play an important role in catalytic activities. There are many common photocatalytic materials, such as TiO_2_ [5], g-C_3_N_4_ [6,7], ZnO [8], and their nanostructure components. According to dimensions, nanophotocatalysts include 0D nanomaterials [9,10], 1D nanotubes [11], 2D nanosheets [12] and 3D aerogel [13,14] materials. Among them, g-C_3_N_4_ is favored due to its suitable band structure, diverse morphology, chemical and thermal stability, low cost and environmental friendliness [15,16,17]. Particularly, g-C_3_N_4_-based aerogels have been widely studied. For example, a 3D porous g-C_3_N_4_/GO (p-CNG) skeleton was constructed via a thermal treatment aided by the template technique. Then, precious metal (Au, Pd, and Pt) cocatalysts were immobilized to the 3D p-CNG skeleton to construct 3D p-CNG-M (Au, Pd and Pt) composite catalysts. Due to the typical 3D porous structure and bonding interaction between g-C_3_N_4_ and GO, 3D p-CNG-M (Au, Pd and Pt) has a large specific surface area and stable physicochemical properties. Meanwhile, precious metal cocatalysts acting as electron acceptors remarkably increase the number of active sites and promote electron–hole separation. Therefore, 3D p-CNG-M catalysts present outstanding enhanced hydrogen evolution reaction activities under simulated solar light [16]. Furthermore, Xu et al. prepared a novel 3D graphene aerogel composite Nb_2_O_5_-g-C_3_N_4_/rGA. The 1D–2D NbNR-CN heterojunction was first prepared via a simple grinding and calcination method. Then, different amounts of NbNR-CN (30%, 60% and 90%) were loaded onto graphene nanosheets via a chemical reduction self-assembly method to obtain the 3D composites Nb_2_O_5_-g-C_3_N_4_/rGA (NbNR-CN/rGA) composites. Compared with g-C_3_N_4_, NbNR and NbNR-CN, NbNR-CN/rGA showed the lowest charge transfer resistance and the highest photocurrent density. Photodegradation experiments showed that the NbNR-CN/rGA composites exhibited remarkably enhanced visible-light photocatalytic activity for the degradation of Rhodamine B (RhB). The removal rate for the 60% NbNR-CN/rGA composite was 94.8% within 100 min. This enhanced photoactivity could be explained by a prolonged lifetime of photo-generated carriers due to the formation of heterojunctions as well as the good electron transfer ability of rGA [11]. On the one hand, aerogels with a 3D network structure can avoid aggregation and provide convenient transfer channels. On the other hand, the block structure of aerogels not only can prevent itself from dispersing in water, but also simplifies the process of operation, collection and separation of materials from water. More importantly, combinations with other photocatalysts to construct g-C_3_N_4_-based aerogel can not only greatly improve the specific surface area, but also expand the spectral utilization range and hinder the rapid recombination of photo-generated electrons and holes. A large number of studies and reports have proven that aerogels have outstanding advantages in adsorption and catalysis due to their massive pores, high catalytic activity, low density and other properties [18,19,20,21]. Carbon dots (CDs), as a new 0D material, are often used as light energy conversion components to construct high-efficiency photocatalysts [12,13,22]. CDs have tunable fluorescence properties, flexible surface states and excellent electron transport channels. Moreover, CDs themselves are also photocatalysts. In previous work, we successfully synthesized novel porous nitrogen-doped carbon dots of (NCDs)@g-C_3_N_4_ composite via a facile hydrothermal approach. The degradation rate of 2.0 wt% NCDs@g-C_3_N_4_ is about twice that of pristine g-C_3_N_4_ at 60 min under the same conditions. In addition, at 120 min, MB was almost degraded completely by 2.0 wt% NCDs@g-C_3_N_4_ with a degradation rate as high as 97.9%, while that of pristine g-C_3_N_4_ was only 47%. Moreover, NCDs were found to have better photocatalytic performance than that of g-C_3_N_4_. At 120 min, the degradation rate of NCDs is 72%. This improved photocatalytic activity can be attributed to the fact that the addition of NCDs not only broadens the photoresponse range, but also suppresses the photo-generated electron and hole recombination of g-C_3_N_4_. Meanwhile, NCDs themselves are also excellent photocatalysts [12]. Furthermore, N-modified carbon dots/graphite carbon nitride (NCDs/g-C_3_N_4_) aerogels were successfully prepared by a simple electrostatic self-assembly method. Among them, NCDs carry a large amount of –NH– from polyethyleneimine (PEI), exhibiting positivity. g-C_3_N_4_ nanosheets exhibit a negative charge. The hydrogen evolution rate of NCDs/g-C_3_N_4_ aerogel can reach 13,499 μ mol h^−1^ g^−1^, which is 1.6 times that of pure g-C_3_N_4_ [14]. Generally speaking, in the process of constructing aerogel, high-molecular polymers such as agar, polyethylene, chitosan, etc., often play a key role [13,23,24].

Herein, the agar, CDs, and g–C_3_N_4_ aqueous solutions were transformed into mixed hydrogels with a 3D network structure by heating–cooling polymerization. The Agar/CDs/g-C_3_N_4_ aerogel was obtained by further freeze drying. Agar can maintain the aerogel structure well and adsorb organic pollutants. Meanwhile, CDs can effectively expand the spectral utilization range of aerogels and reduce the recombination rate of photo-generated electrons and holes. Finally, the Agar/CDs/g-C_3_N_4_ aerogel showed high photocatalytic degradation for AMX, implying that it has enormous potential as a photocatalyst for degrading antibiotic pollutants. The preparation process of aerogel only involves simple agitation and heating and freeze drying, which are very friendly to industrialization. It is also suitable for the preparation of almost all organic aerogels. g-C_3_N_4_ nanosheets are obtained by multiple thermal sintering processes, which is also suitable for extending to other 2D materials, such as graphene. The CDs are prepared through the hydrothermal method, which can be used for the synthesis of almost all nanocrystals/microcrystals. Therefore, the experimental methods are universal, easy to operate, and suitable for large-scale industrial production.

## 2. Results and Discussion

**Figure 1:**
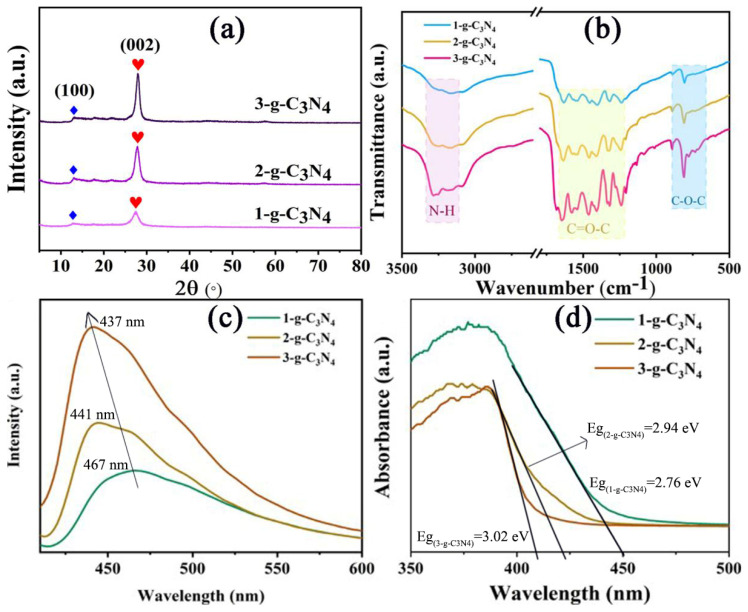
(**a**) The XRD patterns, (**b**) the FTIR spectra, (**c**) the UV–vis absorbance spectra, and (**d**) the PL spectra of 1-g-C_3_N_4_, 2-g-C_3_N_4_ and 3-g-C_3_N_4_.

As shown in Figure S1, multiple thermal sintering is an effective method to obtain g-C_3_N_4_ nanosheets. The 1-g-C_3_N_4_ shows thick micron-scale blocks (Figure S1a). After secondary calcination, the particle size of obtained 2-g-C_3_N_4_ significantly decreased (200 nm–1 µm). What’s more, layered structures of 2-g-C_3_N_4_ can be observed clearly (Figure S1b). 3-g-C_3_N_4_ nanosheets with curled edges were obtained by thermal stripping of 2-g-C_3_N_4_. The particle size of 3-g-C_3_N_4_ is around 200 nm and there is no obvious aggregation (Figure S1c). Ultra-thin 3-g-C_3_N_4_ nanosheets have a larger specific surface area and abundant active sites, which is beneficial for subsequent photocatalytic. The g-C_3_N_4_ nanosheets with relatively complete crystallization and stable luminescence were obtained through the thermal stripping method. As shown in Figure 1a, the XRD results show that g-C_3_N_4_ nanosheets have two characteristic peaks, which are 13.2° and 27.5°, respectively. Among them, the diffraction peak at 27.5° is the strongest, which is the characteristic peak of interlayer stacking of aromatic compounds [20]. The crystal plane index is marked as (002), corresponding layer spacing is d = 0.324 nm, indicating that the g-C_3_N_4_ nanosheets have a layered structure similar to graphite [21]. The other diffraction peak appears at 13.2°, belonging to the characteristic peak of the melon-like substance [25]. The nitrogen pore spacing corresponding to the 3-s-triazine structure is d=0.670 nm, and the crystal plane index is marked as (100) [26]. With the increase in thermal stripping times, the diffraction peak near 27.5° shifts to a higher angle, indicating that the layer spacing of g-C_3_N_4_ becomes smaller. What’s more, with the increase in sintering times, the diffraction peaks of g-C_3_N_4_ become stronger and maintain high symmetry, indicating that the crystallinity of g-C_3_N_4_ nanosheets become more and more complete. In the FTIR spectra of g-C_3_N_4_, the characteristic peaks are mainly distributed in the following three regions, 810 cm^−1^, 1240–1643 cm^−1^, and 3200 cm^−1^ [23,24], which is consistent with the literature reports (Figure 1b). Among them, the peak at 810 cm^−1^ corresponds to the triazine structure. The 1240–1643 cm^−1^ is attributed to a triangular C–N–C structure or bridging C–NH–C unit. The wide band at about 3200 cm^−1^ is the N–H stretching vibration peak, which indicates that there are a lot of amino groups at the edge of g-C_3_N_4_ nanosheets. The thinner of g-C_3_N_4_ nanosheets, the more obvious the signal of amino groups becomes. The PL and UV–vis absorbance spectra display that, with the increase in sintering times, the g-C_3_N_4_ nanosheets are thinner, the layer spacing is smaller, the fluorescence emission is stronger, the luminescence shifts bluer (Figure 1c), and the optical band gap is larger (Figure 1d). The degradations of AMX by g-C_3_N_4_ with different sintering degrees under visible light were studied (Figure S3). It can be seen that the photocatalytic performance gradually increases with the increase in sintering times. Among them, the g-C_3_N_4_ nanosheets sintered three times have the best degradation performance for AMX. The degradation of AMX is basically completed in 40 min, while 1-g-C_3_N_4_ only degrades 50% of AMX. This is due to the fact that thinner nanosheets can provide more reactive sites in the process of photocatalytic oxidation reduction [27]. Therefore, 3-g-C_3_N_4_ was selected and compounded with CDs and agar to construct aerogel in the subsequent experiments.

**Figure 2:**
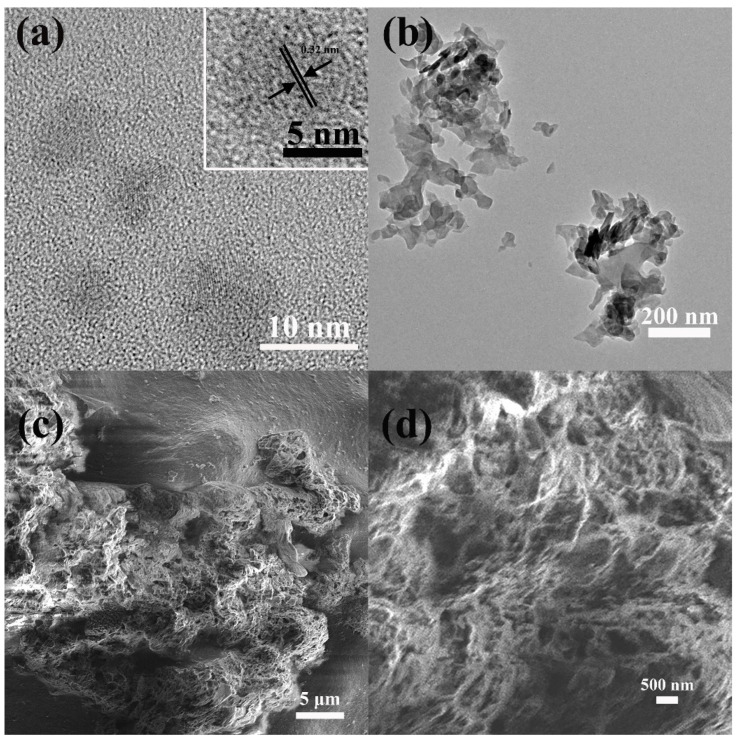
(**a**) The TEM image of CDs, the inset picture is HRTEM image of CDs, (**b**) the TEM image of 3-g-C_3_N_4_, (**c**,**d**) the SEM images of Agar/CDs/g-C_3_N_4_ aerogel.

As shown in Figure 2a, CDs are monodispersed, and their average lateral size determined by TEM is about 6.5 nm with a narrow size distribution (Figure S2). It can be seen that CDs have a certain degree of crystallinity. The high resolution TEM (HRTEM) further proves that the lattice stripe spacing of CDs is 0.32 nm (inset in Figure 2a), corresponding to the (002) crystal plane of graphene [18]. In Figure 2b, the 3-g-C_3_N_4_ nanosheets are about 200 nm. Agar is used as the support skeleton and adsorption module of aerogel, g-C_3_N_4_ is used as the photocatalysis main material, and CDs are used as light energy conversion components. The Agar/CDs/g-C_3_N_4_ aerogel was formed by self-assembly of the three components. The 3D interconnected network stacked by g-C_3_N_4_ and agar has porous, wrinkled, and fluffy microstructures (Figure 2c). The enlarged image in Figure 2d clearly shows that the holes of aerogel are of different sizes and interconnected. The 3D structure of Agar/CDs/g-C_3_N_4_ aerogel effectively prevents the stacking of g-C_3_N_4_ nanosheets. What’s more, the rich pore structure further effectively increases the specific surface area of materials, providing abundant active sites for adsorption and catalytic processes in photocatalytic reactions.

**Figure 3:**
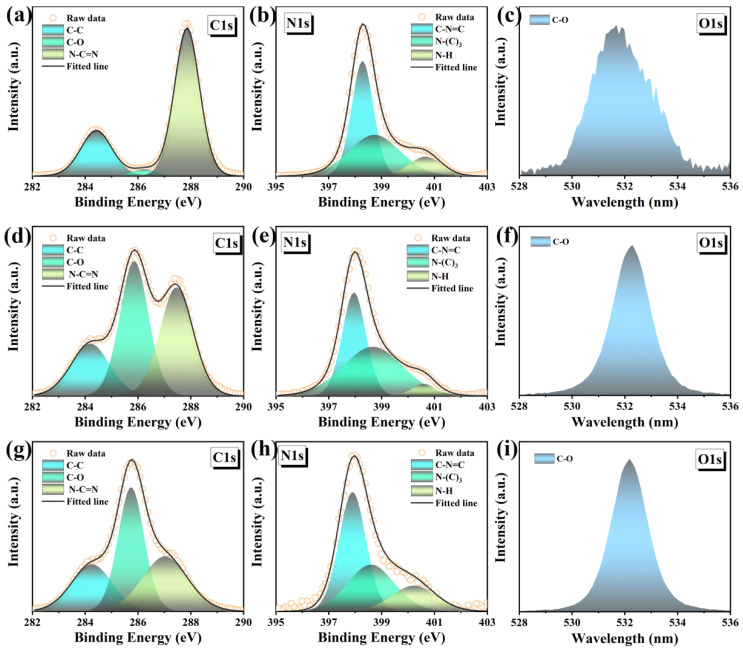
The high-resolution C1s, N1s and O1s XPS spectra of (**a**–**c**) 3-g-C_3_N_4_, (**d**–**f**) Agar/g-C_3_N_4_ and (**g**–**i**) Agar/CDs/g-C_3_N_4_.

The chemical structures of the samples were carefully studied by XPS and FTIR. The g-C_3_N_4_, Agar/g-C_3_N_4_ and Agar/CDs/g-C_3_N_4_ only contain C, N and O three elements (Figure S4). The high-resolution C1s spectra (Figure 3a,d,g) show that the peaks of g-C_3_N_4_, Agar/g-C_3_N_4_ aerogel, and Agar/CDs/g-C_3_N_4_ aerogel can be fitted to three components, belonging to C–C (284.8 eV), C–O (286.45 eV) and N–C=N (288.15 eV), respectively [15,16,17]. It can be seen that the integrated area of the C–O peak in the Agar/g-C_3_N_4_ aerogel is greatly increased (Figure 3d), compared with that of g-C_3_N_4_ (Figure 3a), which is attributed to successful compounding of agar. At the same time, due to the introduction of CDs, the C–C and C–O peaks of Agar/CDs/g-C_3_N_4_ aerogel increased significantly (Figure 3g). As shown in Figure 3b,e,h, the N1s high-resolution spectra show the main structural component at 398.3 eV, which is attributed to the *sp*^2^-bonded nitrogen in the tris-triazine ring (C–N=C) [15], while the weak peak at 398.9 eV is usually attributed to N–(C)_3_ [16]. In addition, the peak at 400.7 eV indicates the presence of amino groups (N–H) [17]. Among them, the Agar/CDs/g-C_3_N_4_ aerogel has the strongest N–H peak (Figure 3h) due to the abundant amino groups on the surface of CDs (Figure S5). Finally, the high-resolution O1s spectra of all samples show the peak at 532.9 eV, corresponding to the C–O bond [28]. A large number of functional groups can serve as active sites for photocatalysis, transferring electrons effectively during the redox reaction process.

**Figure 4:**
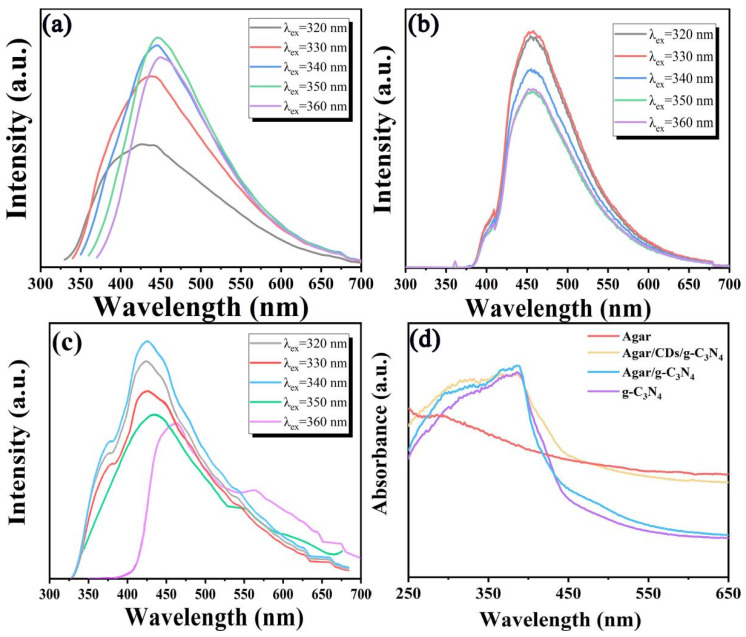
The PL spectra of (**a**) CDs, (**b**) 3-g-C_3_N_4_ and (**c**) Agar/CDs/g-C_3_N_4_ under different excitation wavelength, (**d**) the UV-vis absorbance spectra of Agar, g-C_3_N_4_, Agar/g-C_3_N_4_ and Agar/CDs/g-C_3_N_4_.

In order to explore the optical properties of CDs, 3-g-C_3_N_4_, Agar/g-C_3_N_4_ aerogel and Agar/CDs/g-C_3_N_4_ aerogel, the PL and UV-Vis absorption spectra were recorded. In Figure 4a, it can be observed that CDs have the typical excitation wavelength dependence. The emission peak of CDs is about 410 nm excitated by 320 nm. With the increase in excitation wavelength, the emission peak of CDs gradually redshifts. Meanwhile, the emission intensity of CDs shows first increasing and then decreasing. When the excitation wavelength is 350 nm, the emission peak of CDs is fixed at about 450 nm, and the fluorescence emission intensity of CDs is the highest. In Figure 4b, the emission peak of 3-g-C_3_N_4_ is not affected by the excitation wavelength. Its maximum emission wavelength is 455 nm under 330 nm. Due to the addition of CDs, the emission spectra of Agar/CDs/g-C_3_N_4_ aerogel were significantly enhanced at 330–400 nm and 500–700 nm compared with pure 3-g-C_3_N_4_ (Figure 4c). Moreover, the Agar/CDs/g-C_3_N_4_ aerogel has so wide spectral range that covers the whole visible-light wavelength range (350–700 nm), indicating that visible light can be well utilized by the Agar/CDs/g-C_3_N_4_ aerogel. Generally, the efficiency of light absorption and carrier separation will affect the photocatalytic performance of catalysts [28,29]. Thus the absorption spectra of 3-g-C_3_N_4_, Agar/g-C_3_N_4_ aerogel and Agar/CDs/g-C_3_N_4_ aerogel were explored. As shown in Figure 4d, due to the successful combination of CDs, the absorption intensity of Agar/CDs/g-C_3_N_4_ aerogel was significantly improved after 400 nm. Furthermore, compared with 3-g-C_3_N_4_, the absorption edge of Agar/CDs/g-C_3_N_4_ aerogel is red shifted and its optical band gap is reduced. The narrower optical gap also means that more visible light can be used for photocatalytic activities.

**Figure 5:**
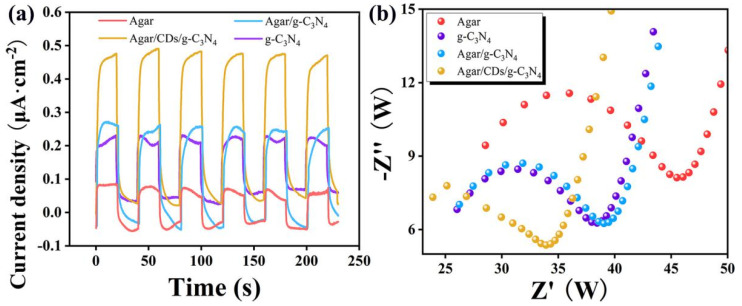
(**a**) Transient photocurrent responses of Agar, Agar/g-C_3_N_4_, 3-g-C_3_N_4_ and Agar/CDs/g-C_3_N_4_ under 300 W Xe-lamp irradiation for six on–off cycles; (**b**) the EIS Nyquist plots of Agar, Agar/g-C_3_N_4_, 3-g-C_3_N_4_ and Agar/CDs/g-C_3_N_4_.

Furthermore, the differences in the photo-generated carriers in a series of samples were studied through photocurrent and impedance tests. As shown in Figure 5a, at the beginning of light illumination, the photocurrent density of each sample rapidly increases. After the end of light illumination, the photocurrent value rapidly decreases again. It indicates that all samples have good light response ability. By switching the laser light six times in a row, all samples show six consecutive stable responses, suggesting the stable output of abundant photo-generated carriers. In fact, high photocurrent density represents a high concentration of photo-generated carriers, indicating good photoelectric conversion ability of samples. Obviously, the photocurrent density of Agar/CDs/g-C_3_N_4_ aerogel is the highest, which is 3.7 times that of agar, 2.4 times that of 3-g-C_3_N_4_, and 1.6 times that of Agar/g-C_3_N_4_ aerogel. What’s more, the EIS measurement is used for further quantitative analysis of the resistance characteristics of Agar, 3-g-C_3_N_4_, Agar/g-C_3_N_4_, and Agar/CDs/g-C_3_N_4_ aerogel in Figure 5b. Generally, the smaller radius of the Nyquist plots corresponds to the faster electron transfer kinetics of the redox reaction and lower charge transfer resistance. The diameter of the Nyquist plot of Agar/CDs/g-C_3_N_4_ aerogel is the smallest. It is proved that, compared to the other three samples, Agar/CDs/g-C_3_N_4_ aerogel has more outstanding charge transfer ability, and excellent charge transfer and separation rates are crucial for improving photocatalytic performance [19].

**Figure 6:**
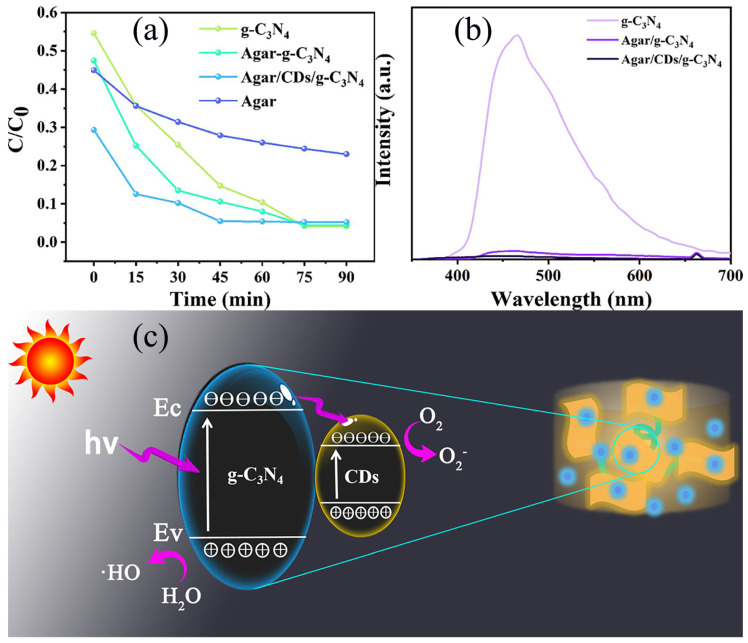
(**a**) The photocatalytic degradation curve of Agar, 3-g-C_3_N_4_, Agar/g-C_3_N_4_ aerogel and Agar/CDs/g-C_3_N_4_ aerogel, (**b**) the PL spectra of 3-g-C_3_N_4_, Agar/g-C_3_N_4_ aerogel and Agar/CDs/g-C_3_N_4_ aerogel, (**c**) the schematic illustration of photocatalytic processes for Agar/CDs/g-C_3_N_4_.

The degradation of AMX by Agar, g-C_3_N_4_, Agar/g-C_3_N_4_ aerogel and Agar/CDs/g-C_3_N_4_ aerogel under visible light irradiation was studied. Before turning on the xenon lamp for photocatalytic experiments, the sample was added to the AMX solution and stirred for 30 minutes in the dark to achieve adsorption–desorption equilibrium. As shown in Figure 6a, when the adsorption equilibrium is reached at 0 min, the removal performance of all aerogel samples for AMX is higher than that of pure g-C_3_N_4_ due to the fluffy porous structure formed by agar. On the one hand, the 3D structure of aerogel can provide a convenient transfer channel for the adsorption and in situ degradation of AMX. On the other hand, it can avoid the aggregation of g-C_3_N_4_ and prevent them from dispersing in water, greatly simplifying the collection and separation of g-C_3_N_4_ from water. After light irradiation, the degradation efficiency of agar is very low, because pure agar has little photocatalytic performance. Agar/g-C_3_N_4_ aerogel shows a stronger removal efficiency compared with g-C_3_N_4_. The reason is the effect of adsorption from agar. More importantly, after the successful recombination of CDs, the photocatalytic ability of Agar/CDs/g-C_3_N_4_ was further improved. AMX was almost completely degraded by Agar/CDs/g-C_3_N_4_ after 45 min of illumination, while it takes 75 min for Agar/g-C_3_N_4_ aerogel and g-C_3_N_4_ to completely remove AMX. It is not only due to the high adsorption ability brought by agar, but also thanks to the introduction of CDs. Compared with g-C_3_N_4_, the fluorescence emission intensity of Agar/g-C_3_N_4_ aerogel decreases sharply, indicating that it has higher carrier separation efficiency (Figure 6b). The reason is the unique 3D interconnected porous structure of aerogel hinders the recombination of electrons and holes. With the successful combination of CDs, the fluorescence emission intensity of Agar/CDs/g-C_3_N_4_ aerogel further decreased, indicating that CDs inhibited the rapid recombination of photo-generated electrons and holes. In a word, the Agar/CDs/g-C_3_N_4_ aerogel has the best carrier transport efficiency, which is consistent with the analysis results of photocurrent and impedance. It is because photo-generated electrons and holes are transferred from the g-C_3_N_4_ surface to the surface of CDs under visible light irradiation. Then, free groups (such as O^2−^ and ·HO) are generated for the degradation of AMX in the water and oxygen environment (Figure 6c). AMX molecules and intermediates might be oxidized into other small molecules and ultimately mineralized into H_2_O and CO_2_. Most of the photodegradation intermediates of AMX are harmless and avirulent [27,28]. After considering the materials (including glucose, agar, melamine, and deionized water), consumables (including dialysis bags and test tubes), and energy costs, the cost of preparing 10 g Agar/CDs/g-C_3_N_4_ aerogel was calculated at about 17.55 RMB. The commercial P25 photocatalyst is priced at 28 RMB per 10 g. More importantly, Agar/CDs/g-C_3_N_4_ aerogel can degrade AMX in 45 minutes, while the degradation of commercial P25 for AMX takes 180 min [30]. Thus, Agar/CDs/g-C_3_N_4_ aerogel has been proven to have good application prospects and commercial value.

## 3. Conclusions

In summary, Agar/CDs/g-C_3_N_4_ aerogel photocatalyst was prepared by simple heating, compounding and freeze-drying with agar as the adsorption module and CDs as light energy conversion components. Agar/CDs/g-C_3_N_4_ aerogel has a larger specific surface area, richer functional groups, wider spectra range, higher photocurrent density and better carrier migration and separation efficiency compared with other samples, therefore, it has the best photocatalytic degradation performance. After 45 min of illumination, AMX was almost completely degraded by Agar/CDs/g-C_3_N_4_ aerogel. It is not only attributed to the fact that the aerogel interconnected porous structure can provide more active sites for redox reaction in photocatalytic activity, but also due to the wider visible light utilization range and higher carrier separation efficiency. It is a good idea to realize the design of aerogel that can efficiently photocatalytic degrade antibiotic pollutants by using the effects of each component in the ternary system.

## 4. Materials and Methods

### 4.1. Materials

Ethanol (CH_3_CH_2_OH, ≥99.5%), melamine (C_3_H_6_N_6_, 99%), Agar ((C_12_H_18_O_9_)_n_, high gel strength (1000–1200 g/cm^2^)), and glucose (C_6_H_12_O_6_, 96%) Amoxicillin (C_16_H_19_N_3_O_5_S, ≥98%) were purchased from Aladdin Chemistry Co., Ltd. (Shanghai, China). Deionized water was used during the experiment.

### 4.2. Methods

#### 4.2.1. Preparation of g-C_3_N_4_

First, the yellow bulk g-C_3_N_4_ was prepared by the classical thermal sintering method. Melamine (1.5 g) was calcined in a muffle furnace at a heating rate of 2.5 °C/min at 550 °C for 5 h in an air atmosphere. The obtained yellow block was ground to obtain 1-g-C_3_N_4_. Then, the 1-g-C_3_N_4_ was thermally stripped two times to obtain fluffy milky white 3-g-C_3_N_4_ flakes. Specifically, 500 mg 1-g-C_3_N_4_ powder was heated at 530 °C for 3 h in an air atmosphere to obtain the pale yellow powder of 2-g-C_3_N_4_. Then 2-g-C_3_N_4_ was taken out, ground and heated at 500 °C for 1 h in an air atmosphere to obtain the fluffy milky white 3-g-C_3_N_4_.

#### 4.2.2. Preparation of CDs

0.5 g of glucose was dissolved in 24 mL 1:1 ethanol aqueous solution, and the mixture was transferred to a high-pressure vessel sealed with polytetrafluoroethylene and heated at 200 °C for 170 min. After cooling to room temperature, the obtained solution was transferred to a dialysis bag with 1000 Da for 24 h dialysis, changing water 3–4 times during the process. After removing solvents and further freeze drying, the obtained brown powder is CDs.

#### 4.2.3. Preparation of Aerogels

Agar/CDs/g-C_3_N_4_ aerogel is prepared by a simple method. Specifically, 0.4 g 3-g-C_3_N_4_ powder, 0.1 g agar and 0.03 g/mL CDs solution are added to 25 mL of deionized water successively and stirred for 1.5 h. Then the mixed solution was heated at 90 °C in a constant temperature water bath for 10 min, and cooled it naturally to room temperature to obtain agar/CDs/g-C_3_N_4_ hydrogel. Finally, the hydrogel was freeze dried for more than 36 h to obtain Agar/CDs/g-C_3_N_4_ aerogel.

The preparation method of Agar/g-C_3_N_4_ aerogel is to remove the CDs solution, and then prepare it under the same conditions and steps. The specific amounts of materials are 0.4 g of 3-g-C_3_N_4_ powder, 0.1 g of agar, and 25 mL of deionized water.

#### 4.2.4. Cost Calculation for Agar/CDs/g-C_3_N_4_ Aerogel

Taking the preparation of 10 g Agar/CDs/g-C_3_N_4_ aerogel as an example. The preparation of 10 g of Agar/CDs/g-C_3_N_4_ requires 3.2 g of g-C_3_N_4_, 6 g of agar (3 RMB), and 0.8 g of CDs; 10 g (1.8 RMB) melamine is required to prepare 3.2 g g-C_3_N_4_; 1 g of glucose (0.15 RMB), 12 mL of ethanol (0.4 RMB) and 12 mL of deionized water (0.2 RMB) are needed to prepare 0.8 g CDs. The costs of consumables (dialysis bags, test tubes, etc.) and energy (electricity) are approximately 12 RMB. Therefore, the preparation cost of 10 g of Agar/CDs/g-C_3_N_4_ aerogel is 17.55 RMB.

## 5. Characterizations

The crystalline phase of samples was identified by X-ray powder diffractometer (XRD, D/max–2200, Rigaku, Tokyo, Japan) with Cu Kα radiation (λ = 1.54178 Å,) in the 2θ range from 5° to 80° at room temperature, and the tube voltage and current of the instrument were operated at 40 kV and 30 mA, respectively. A transmission electron microscope (TEM) (FEI, TF20, Hillsboro, OR, USA) and a field emission scanning electron microscope (Zeiss Gemini SEM 300, Jena, Germany) were employed to observe morphologies of CDs, g-C_3_N_4_ and Agar/CDs/g-C_3_N_4_ aerogel. Fourier transform infrared (FT–IR) spectra were recorded on a Nicolet Nexus 670 FTIR spectrometer (Nicolet Instruments, Madison, WI, USA) within KBr slices in the 4000–400 cm^−1^ range. X-ray photoelectron spectroscopy (XPS) (Thermo Fisher Scientific K–Alpha^+^, East Grinstead, UK) was performed using a monochromatic Al Kα with an energy of 1486.6 eV. When the pressure of sample chamber was less than 2.0 × 10^−7^ mba, the sample was sent to the analysis room. The spot size, the working voltage and the filament current are 400 µm, 12 kV and 6 mA, respectively. The energy of full spectrum scanning mode is 150 eV and its step size is 1 eV. The energy of narrow spectrum scanning mode is 50 eV and its step size is 0.1 eV. Using a white Al_2_O_3_ disk as the background, the UV–vis absorption spectra of samples were recorded on a Hitachi UH-3900 spectrophotometer (Hitachi, Tokyo, Japan) via integrating sphere mode. The photoluminescence (PL) spectra were obtained on an FLS1000 (Edinburgh Instruments Ltd., Livingston, UK) fluorescence spectrometer with a Xe lamp. The photocatalytic experimental is similar to reference [30]. Specifically, photocatalytic activity tests were evaluated via the photocatalytic degradation of AMX under visible light using a 500 W xenon lamp as the only light source. The experiment was carried out in an XQ500 W photochemical reactor (Zuo-Le Instrument Co., Ltd., Shanghai, China). For each experiment, a 50 mg photocatalyst was added to 100 mL AMX solution at a concentration of 3 mg/L. That is the mass ratio of photocatalyst to AMX in the experiment was 167. Prior to light irradiation, the reaction solution was agitated in the dark for 30 min to obtain adsorption equilibrium for AMX on the photocatalyst. During the photocatalytic process, 4 mL samples were extracted every 30 min and the 3 mL of supernatant was separated via centrifuging at 8000 rpm/s. Each sample was catalysed for 90 min. The absorbance of each group of samples was measured by a UV spectrophotometer to calculate the photocatalytic efficiency. The formula is as follows:

P = C/C_0_

C and C_0_ were the UV–vis absorption intensity of uncatalyzed AMX at each sampling and initial catalysis, respectively.

## Data Availability

The data presented in this study are available on request from the corresponding author. The data are not publicly available due to part of the data undergoing in-depth research.

## Supplementary Materials

The following supporting information can be downloaded at https://www.mdpi.com/article/10.3390/gels10020100/s1, Figure S1: the SEM images of (a) 1-g-C_3_N_4_, (b) 2-g-C_3_N_4_ and (c) 3-g-C_3_N_4_; Figure S2: particle size distribution diagram of the CDs; Figure S3: the photocatalytic degradation curve of g-C_3_N_4_; Figure S4: the survey XPS spectrum of (a) g-C_3_N_4_, (b) Agar/g-C_3_N_4_ aerogel and (c) Agar/CDs/g-C_3_N_4_ aerogel; Figure S5: the FTIR spectrum of CDs.
