# Supplementary material for: Aerogel for Highly Efficient Photocatalytic Degradation
Source: Gels. 2024 Jan 26;10(2):100. doi: 10.3390/gels10020100 (PMC10888021; doi:10.3390/gels10020100)
Supplement: Supplementary file 1 [file gels-10-00100-s001.zip › gels-2762439-supplementary.pdf]

Supporting Information

Aerogel for Highly Efficient Photocatalytic Degradation

Xue-Chun Yang [1], Jing-Tai Zhao [2]*

[1] School of Environmental and Chemical Engineering, Shanghai University, Shanghai 200444, P. R. China
[2] School of Materials Science and Engineering, Guilin University of Electronic Technology, Guilin 541004, P. R. China

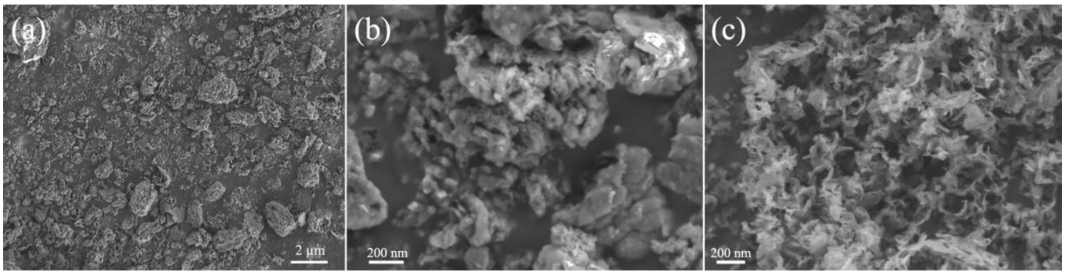

Figure S1. The SEM images of (a) 1-g-C_3N_4, (b) 2-g-C_3N_4 and (c) 3-g-C_3N_4.

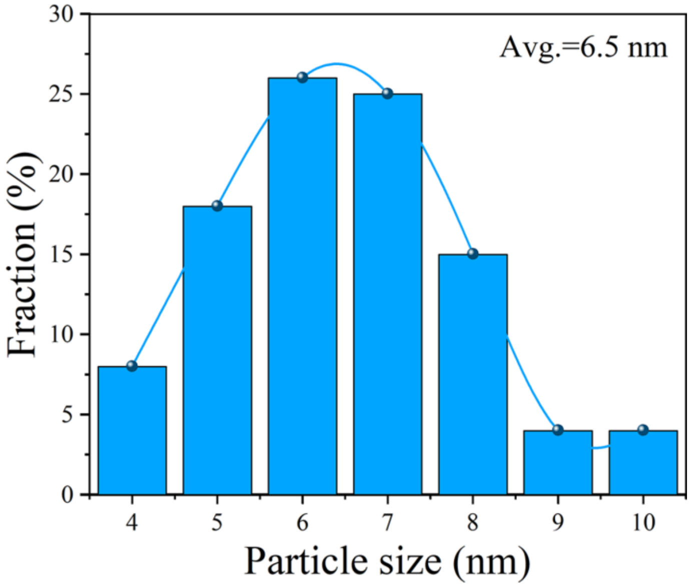

Figure S2. Particle size distribution diagram of the CDs.

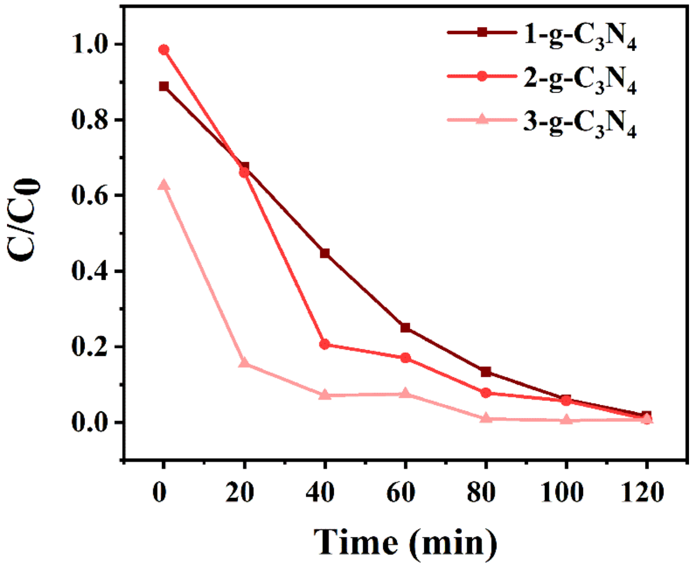

Figure S3. The photocatalytic degradation curve of g-C_3N_4.

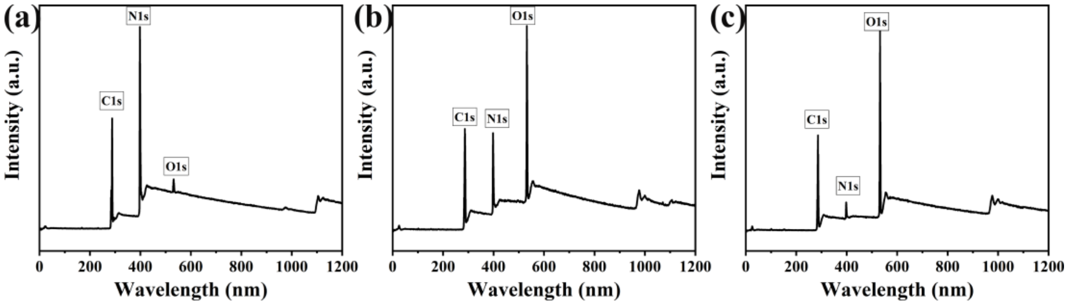

Figure S4. The survey XPS spectrum of (a) g-C_3N_4, (b) Agar/g-C_3N_4 aerogel and (c) Agar/CDs/g-C_3N_4 aerogel.

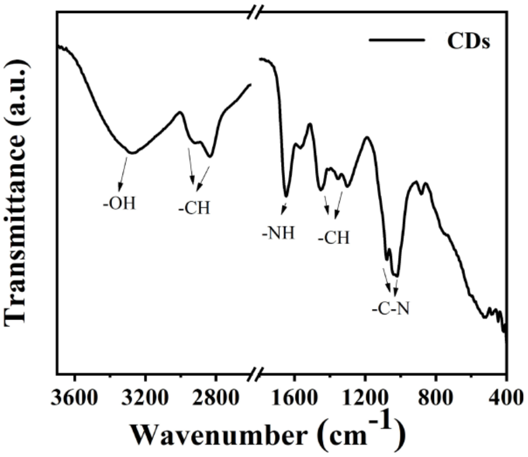

Figure S5. The FTIR spectrum of CDs.
